# A Path Tracking Strategy for Car Like Robots with Sensor Unpredictability and Measurement Errors

**DOI:** 10.3390/s20113077

**Published:** 2020-05-29

**Authors:** Madan Mohan Rayguru, Mohan Rajesh Elara, Balakrishnan Ramalingam, M. A. Viraj J. Muthugala, S. M. Bhagya P. Samarakoon

**Affiliations:** ROAR Lab, Engineering Product Development, Singapore University of Technology and Design, Singapore 487372, Singapore; madan_rayguru@sutd.edu.sg (M.M.R.); rajeshelara@sutd.edu.sg (M.R.E.); viraj_jagathpriya@sutd.edu.sg (M.A.V.J.M.); bhagya_samarakoon@mymail.sutd.edu.sg (S.M.B.P.S.)

**Keywords:** path tracking, measurement error, robust estimation

## Abstract

This work is inspired by motion control of cleaning robots, operating in certain endogenous environments, and performing various tasks like door cleaning, wall sanitizing, etc. The base platform’s motion for these robots is generally similar to the motion of four-wheel cars. Most of the cleaning and maintenance tasks require detection, path planning, and control. The motion controller’s job is to ensure the robot follows the desired path or a set of points, pre-decided by the path planner. This control loop generally requires some feedback from the on-board sensors, and odometry modules, to compute the necessary velocity inputs for the wheels. As the sensors and odometry modules are prone to environmental noise, dead-reckoning errors, and calibration errors, the control input may not provide satisfactory performance in a closed-loop. This paper develops a robust-observer based sliding mode controller to fulfill the motion control task in the presence of incomplete state measurements and sensor inaccuracies. A robust intrinsic observer design is proposed to estimate the input matrix, which is used for dynamic feedback linearization. The resulting uncertain dynamics are then stabilized through a sliding mode controller. The proposed robust-observer based sliding mode technique assures asymptotic trajectory tracking in the presence of measurement uncertainties. Lyapunov based stability analysis is used to guarantee the convergence of the closed-loop system, and the proposed strategy is successfully validated through numerical simulations.

## 1. Introduction

In the recent past, wheeled mobile robots have played a critical role in automating various cleaning and sanitation tasks [1,2]. The importance cannot be stressed more in this pandemic situation. Not only the cleaning robots provide isolation from hazardous and infectious environments, but also they are much more efficient in the overall performance. A cleaning robot generally consists of a base platform with multiple wheels, which may be omnidirectional depending upon applications. The wheeled base platform can carry a versatile set of cleaning modules for different types of cleaning/sanitation requirements. For example, a Human Service Robot (HSR) can have multiple manipulators, which can be fitted with brushes, vacuum pumps, rollers, etc. Similarly, the base platform of a floor cleaning robot may have sweeping brushes, bin bags, etc.

The last two decades have given rise to the development of many types of cleaning robots, depending on the required functionality. The authors of [3] presented a pavement cleaning platform called Panthera, whose width can change according to static and dynamic environments. The design and development of a reconfigurable floor cleaning robot h-Tetro are discussed in [4]. The drain maintenance and sanitation tasks are automated by a four-legged robot with wheels [5]. The HSR platforms are predominantly used for door cleaning, table scrubbing, lift panel sanitation etc.

Even though the cleaning robots discussed above vary in their shape, structure, and functionality, they have one common thing, which is a wheeled base platform. The number of wheels and their types are decided by the range of motion required for the task. An outdoor cleaning robot may require three or four wheels for its movement [6,7], whereas a small indoor cleaning robot may run on two active wheels [8]. The HSR type of robots are fitted with omnidirectional wheels for their movement [9]. Certain reconfigurable robots may contain one wheel in each unit [10,11]. Most of vertical robots also require magnetic or suction wheels for their movement [12].

Given a particular type of platform, the overall task is divided into many sub-steps. For example, a door cleaning robot should first detect the doors in an indoor environment, followed by reaching that particular position avoiding any static or dynamic obstacles [9,13]. Given an instantaneous position and a goal position, the path planning task can be carried out using many techniques. Once a reference path is generated, the motion control algorithm should be able to follow that path without any issues. A motion control policy takes the reference path and sensor measurements as its input, and provide the required input velocity/torque to the motors governing the wheels.

The motion control for wheeled mobile robots (WMRs) has received considerable attention from the research community due to its importance in the efficient completion of the required task. Numerous linear and nonlinear control laws are proposed for trajectory following and posture stabilization of WMR [14,15]. Sometimes, the mathematical model is transformed into a polar coordinate form to simplify the design procedure [16]. Some popular approaches for such tasks are PID control, feedback linearization, backstepping design, linear quadratic regulator (LQR), model predictive control, etc., [17,18]. The disturbances and uncertainties present in the robot are tackled by robust [19,20] or adaptive [15] control techniques.

All the control techniques require the measurements of the state variables, that define the mathematical model of the robot. As the measurements are carried out through sensors and odometry module, full state measurements always add to the cost and complexity of the electronics module of a robot. If some measurements are not available, then output feedback control laws [21] or estimation based control strategies [22,23] are used. The static and dynamic feedback techniques are very useful in terms of practical viewpoint.

It is comparatively easy to get the position measurements, compared to the heading angle, which may require additional cost for gyroscope and IMU. Moreover, a relative coordinate (discrepancy between actual robot posture, and desire posture) can be easily obtained in most of the practical applications by using vision based data or LIDAR/RADAR. However, the outputs of sensor modules and odometry modules are not very accurate inside the indoor environment. The sensor unpredictability may result from various noise and environmental factors. The odometry section may be affected by quantization errors and the accumulation of errors due to dead-reckoning. If the data used for the control module may not point to the exact posture/position of the robot, then they may lead to an inaccurate following of the desired trajectory. Hence, it becomes important to consider these issues while designing control laws for robots.

Many designs of robot tracking controllers for dealing with the absence of heading angle measurement could be found in literature [22]. However, most of the estimation techniques assume the available position measurements are disturbance free. Moreover, the standard designs like PID, output feedback linearization, dynamic feedback linearization [15] may not provide a desired closed-loop performance in the presence of measurement uncertainties. The absence of heading angle information and the presence of measurement uncertainties are the motivation behind this paper. To solve both the problems simultaneously, the work proposes an observer-based robust dynamic feedback linearization for trajectory tracking control in WMR. The dynamic feedback linearization requires the computation of a Jacobian type matrix comprising of trigonometric functions of heading angle, which is unavailable for measurement. Unlike the earlier observer designs, the trigonometric functions are directly estimated in the presence of uncertainties in position measurements. The dynamic feedback linearizing controller is made robust by exploiting the sliding mode technique. The closed-loop stability of the robust observer and sliding mode controller is analysed using Lyapunov technique. The proposed technique assures asymptotic trajectory tracking for car-like robots, whose validation is proved using numerical simulations.

The mathematical model of the robot is described in Section 2. The method proposed for estimating the heading angle is given in Section 3. Section 4 describes the robust control law of the system. The simulation results are analyzed and discussed in Section 5 for the validation. Finally, Section 6 concludes the work.

## 2. Mathematical Model

This paper considers a four-wheel driven car-like robot for deriving trajectory tracking control law. The mathematical model of the robot is derived using two assumptions, similar to [24].

(i) There is no lateral slip while in motion.
(ii) The robot motion platform does not have any flexible parts.

**Figure 1:**
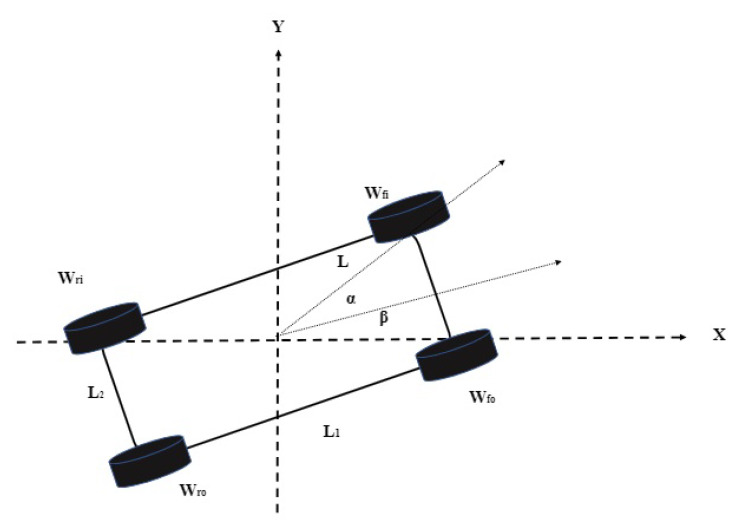
Schematic of the robot.

The geometric relation between the robot center of gravity (COG), and the wheel centers can be observed from Figure 1 (the origin coincides with COG). The pose of the robot at any moment can be defined by $p={\left[x_{r},y_{r},\beta \right]}^{T}$, where β is the heading of the robot with respect to the local coordinate frame. The distance between the front and rear wheels is $L_{1}$, whereas $L_{2}$ represents the width of the robot. All the four wheels ($W_{fo},W_{fi},W_{ro},W_{ri}$) are at a distance *L* from the robot COG. The parameter α denotes the angle between the line of sight of the robot (through COG) and each wheel. Given the position ($x_{o},y_{o}$) of the COG, the longitudinal and transversal position of each wheel can be described below.

(1) $$\begin{array}{cc} \; & W_{fi}\left(x,y\right)=x_{o}+Lcos\left(\alpha +\beta \right),y_{o}+Lsin\left(\alpha +\beta \right)\\ \; & W_{fo}\left(x,y\right)=x_{o}+Lcos\left(-\alpha +\beta \right),y_{o}+Lsin\left(-\alpha +\beta \right)\\ \; & W_{ri}\left(x,y\right)=x_{o}+Lcos\left(\pi -\alpha +\beta \right),y_{o}+Lsin\left(\pi -\alpha +\beta \right)\\ \; & W_{ro}\left(x,y\right)=x_{o}+Lcos\left(\pi +\alpha +\beta \right),y_{o}+Lsin\left(\pi +\alpha +\beta \right) \end{array}$$

The lateral slip of the vehicle is neglected. Hence, the resulting constraint

(2) $${\dot{x}}_{o}sin\left(\beta \right)+{\dot{y}}_{o}cos\left(\beta \right)=0$$

is a nonholonomic one. Similar constraints for the four wheels can be derived by exploiting the wheel positions and their steering angles.

### 2.1. Differential Steering

A car-like robot with independent steering at each wheel can be driven in various ways. As the focus of the paper is to study the effect of measurement and odometry errors on trajectory tracking, we have chosen the Ackermann-Jeantand methodology. In this technique, the outer wheels ($W_{fo},W_{ro}$) have the same speed, and the inner wheel ($W_{fi},W_{ri}$) velocities are also kept the same. This model is also called the reverse differential technique, where the inner and outer wheel speeds are derived as:

(3) $$\begin{array}{cc} \; & v_{in}=v\left(1-\frac{2L_{2}}{L_{1}}tan\left(\beta \right)\right)\\ \; & v_{out}=v\left(1+\frac{2L_{2}}{L_{1}}tan\left(\beta \right)\right)\\ \; & \omega_{in}=\omega \left(\frac{L_{1}}{L_{1}-L_{2}tan\left(\beta \right)}\right)\\ \; & \omega_{out}=\omega \left(\frac{L_{1}}{L_{1}+L_{2}tan\left(\beta \right)}\right) \end{array}$$

where *v* and ω are linear and angular velocities of robot COG respectively.

The forward kinematics of the simplified robot model can be described as:

(4) $$\dot{p}=\left[\begin{array}{c} \dot{x}\\ \dot{y}\\ \dot{\beta } \end{array}\right]=\left[\begin{array}{cc} cos(\beta ) & 0\\ sin(\beta ) & 0\\ 0 & 1 \end{array}\right]\left[\begin{array}{c} v\\ \omega \end{array}\right]$$

### 2.2. Dynamic Feedback Linearization

The trajectory tracking (path following) problem can be defined as [25]: Given a set of desired time-varying coordinate ($x_{d}\left(t\right),y_{d}\left(t\right)$), derive a pair of admissible control input (v,ω) such that the robot can follow the desired path from any arbitrary position (x,y), with minimum/zero discrepancies. So the controlled variables for this problem are *x* and *y*.

(5) $$\left[\begin{array}{c} \dot{x}\\ \dot{y} \end{array}\right]=\left[\begin{array}{cc} cos(\beta ) & 0\\ sin(\beta ) & 0 \end{array}\right]\left[\begin{array}{c} v\\ \omega \end{array}\right]$$

From (5), it can be observed that the control input ω does not explicitly effect ($\dot{x},\;\dot{y}$). However, it can affect them implicitly through the heading angle β. As there exists a singularity in the input matrix, it is not possible to satisfy controllability condition, which is critical for controller design. One of the celebrated ways to circumvent this issue, is to use a dynamic feedback linearization method. The dynamic feedback linearization technique for nonlinear systems, search for a dynamic controller whose output can act as the input for the robot.

For this purpose, consider an integrator of the form

(6) $$\dot{\zeta }=f,\;\;v=\zeta$$

where *f* will be decided later. This integrator acts as a dynamic compensator whose output ζ will act as the control input *v*. If the positions (x,y) are differentiated twice, one may derive:

(7) $$\left[\begin{array}{c} \ddot{x}\\ \ddot{y} \end{array}\right]=\left[\begin{array}{cc} cos(\beta ) & -\zeta sin(\beta )\\ sin(\beta ) & \zeta cos(\beta ) \end{array}\right]\left[\begin{array}{c} f\\ \omega \end{array}\right]=\Theta U$$

where $U=\left[f,\omega \right]\in R^{2}$ is the transformed control input pair, and Θ is the new input matrix.

The trajectory tracking problem for the robot modeled as (4) is converted to the problem of determining an admissible *U* for the mathematical model given in (7). A simple inversion control law

(8) $$U=\Theta^{-1}\left(\xi \right)$$

transforms the system (7) into a linear system of the form:

(9) $$\left[\begin{array}{c} \ddot{x}\\ \ddot{y} \end{array}\right]=\xi$$

where $\xi ={\left[\xi_{1},\xi_{2}\right]}^{T}$ is the new control input. A simple proportional-differential (PD) type control law for ξ can solve the tracking problem for (7).

**Remark** **1.** *The control problems for the wheeled mobile robots can be broadly categorized into trajectory tracking and posture stabilization problems. For a four-wheeled robot with independent driving and steering, the posture stabilization problem can be divided into two steps. The robot should first go to a desired position ($x_{d},y_{d}$), and then the steering motors can complete the orientation task. Hence, a trajectory tracking controller with a PID for steering can complete this task.*

The above control law assumes that the measurements for x,y,β are available and accurate. As this is not the case in many practical applications, the control law should be able to deal with it.

It is assumed that the position measurements for *x* and *y* are available but corrupted by some unknown but bounded disturbances. It should be noted that the implementation of dynamic feedback linearization the control law $U=\Theta^{-1}\xi$ requires an explicit calculation of the input matrix Θ. As the measurement for the heading angle β is unavailable, it is not possible to use the control law directly. The following subsection describes an approach to estimate the matrix Θ.

## 3. Estimation of Θ

The matrix Θ comprises of the known variable ζ=v, and the unknown terms cos(β),sin(β). One can estimate the heading angle β, and derive the unknown terms. However, it is easier to estimate the unknown terms directly without bothering about the heading angle measurement. This approach is also advantageous in cases where the measurement for the heading angle is not accurate. For the purpose of designing an observer, define

(10) $$p_{1}=x,\;p_{2}=y,\;p_{3}=sin\left(\beta \right),\;p_{4}=cos\left(\beta \right).$$

The dynamics of these variables can be written as:

(11) $$\begin{array}{cc} \; & {\dot{p}}_{1}=\zeta p_{4}\\ \; & {\dot{p}}_{2}=\zeta p_{3}\\ \; & {\dot{p}}_{3}=\omega p_{4}\\ \; & {\dot{p}}_{4}=-\omega p_{3} \end{array}$$

The states $p_{1}$ and $p_{2}$ are available for measurement, even though they are corrupted. These data may be corrupted due to random noise, and some deterministic errors like quantization error during implementation. To remove the random noise, the measurements are passed through Kalman filter, and the technique is widely documented. If the computation power is limited, one may use a simple low pass filter to remove any high-frequency noise. Here, it is assumed that the measurements are already passed through an adequate filter to remove any random high-frequency noise. The filtered measurements can be represented as:

(12) $$p_{x}=p_{1}+n_{x},\;\;p_{y}=p_{2}+n_{y}$$

where $n_{x}$ and $n_{y}$ are measurement uncertainties in *x* and *y* respectively. The following assumptions are important for practical implementation.

**Assumption** **1.** *There exist two positive constants $c_{1},\;c_{2}$ such that the measurement uncertainties and their derivatives satisfy,*

(13) $$\left|\right|n_{x}\left|\right|\leq c_{1},\;||n_{y}\left|\right|\leq c_{1},\;||{\dot{n}}_{x}\left|\right|\leq c_{2},\;||{\dot{n}}_{y}\left|\right|\leq c_{2}.$$

Assumption I is automatically satisfied in most of the practical scenarios. By design, the sensors measuring the position can not give an unbounded value. Moreover, the errors due to sensor inaccuracies and dead-reckoning are always bounded. The rate of change of these errors $({\dot{n}}_{x},{\dot{n}}_{y}$) can be large in case of a sudden change/fault and produce a high-frequency noise, but they can be eliminated by a suitable filter. The controller needs to act on a finite part of them, which can not be filtered. So restricting $({\dot{n}}_{x},{\dot{n}}_{y}$) is not conservative in practical situations.

**Assumption** **2.** *The linear velocity v of the robot is always positive except at the origin of the error coordinates.*

Assumption II means, the velocity of the vehicle can not be zero apart from the origin, or when tracking error is zero. This assumption can be satisfied by ensuring the minimum velocity to be a very small non-zero value unless the robot is at the origin [15].

A reduced order observer is constructed using $p_{x},\;p_{y}$ as:

(14) $$\begin{array}{cc} \; & {\dot{\bar{p}}}_{3}=\omega {\hat{p}}_{4}-l\left(t\right)\left(\zeta {\hat{p}}_{3}\right)\\ \; & {\dot{\bar{p}}}_{4}=-\omega {\hat{p}}_{3}-l\left(t\right)\left(\zeta {\hat{p}}_{4}\right)\\ \; & {\hat{p}}_{3}={\bar{p}}_{3}+l\left(t\right)p_{x}\\ \; & {\hat{p}}_{4}=-{\bar{p}}_{4}+l\left(t\right)p_{y} \end{array}$$

where ${\bar{p}}_{3},\;{\bar{p}}_{4}$ can be regarded as intermediate variables, and ${\hat{p}}_{3},\;{\hat{p}}_{4}$ are the estimates for the unknown terms sin(β),cos(β). The time varying observer gain l(t) is selected as:

(15) $$l\left(t\right)=\left(l_{o}-l_{a}\right)exp\left(-l_{n}t\right)+l_{a}$$

where the design scalars $l_{o},l_{a}$ and $l_{n}$ are positive constants, which need to be tuned for better performance. It should be noted that the observer gain is always positive ∀,t≥0, and satisfies

(16) $$l_{a}\leq l\left(t\right)\leq l_{o},$$

for the choice $l_{o}>l_{a}$. The estimate for the matrix Θ can be computed as:

(17) $$\hat{\Theta }=\left[\begin{array}{cc} {\hat{p}}_{4} & -\zeta {\hat{p}}_{3}\\ {\hat{p}}_{3} & \zeta {\hat{p}}_{4} \end{array}\right]$$

### Convergence Analysis of the Observer

It is important to assure the convergence of the estimates ${\hat{p}}_{3}$ and ${\hat{p}}_{4}$ to their true values. For this sake define two error variables

(18) $${\tilde{p}}_{3}=p_{3}-{\hat{p}}_{3},\;{\tilde{p}}_{4}=p_{4}-{\hat{p}}_{4}$$

Exploiting the Equation (11), the measurements (12) and the observer dynamics (14), the dynamics of estimation error can be derived as:

(19) $$\begin{array}{cc} \; & {\dot{\tilde{p}}}_{3}=\omega {\tilde{p}}_{4}-l\left(t\right)\left(\zeta {\tilde{p}}_{3}\right)-l\left(t\right)\left({\dot{n}}_{x}\right)\\ \; & {\dot{\tilde{p}}}_{4}=-\omega {\tilde{p}}_{3}-l\left(t\right)\left(\zeta {\tilde{p}}_{4}\right)-l\left(t\right)\left({\dot{n}}_{y}\right) \end{array}$$

The convergence can be analyzed for two cases.

Case 1: First consider the case when the measurement uncertainties $n_{x},n_{y}$ are slowly varying. For this case

(20) $${\dot{n}}_{x}={\dot{n}}_{y}\approx 0.$$

The estimation error dynamics becomes,

(21) $$\begin{array}{cc} \; & {\dot{\tilde{p}}}_{3}=\omega {\tilde{p}}_{4}-l\left(t\right)\left(\zeta {\tilde{p}}_{3}\right)\\ \; & {\dot{\tilde{p}}}_{4}=-\omega {\tilde{p}}_{3}-l\left(t\right)\left(\zeta {\tilde{p}}_{4}\right)\\ \; & \Rightarrow \left[\begin{array}{c} {\dot{\tilde{p}}}_{3}\\ {\dot{\tilde{p}}}_{4} \end{array}\right]=\left[\begin{array}{cc} -l(t)\zeta & \omega \\ -\omega & -l(t)\zeta \end{array}\right]\left[\begin{array}{c} {\tilde{p}}_{3}\\ {\tilde{p}}_{4} \end{array}\right] \end{array}$$

As ζ=v and l(t)>0,∀t≥0 from (16), it can be concluded that ζl(t)>0,∀t≥0.

Consider a candidate Lyapunov function

(22) $$V_{o}=\frac{1}{2}\left[{\tilde{p}}_{3},{\tilde{p}}_{4}\right]{\left[{\tilde{p}}_{3},{\tilde{p}}_{4}\right]}^{T}.$$

The time derivative of this function along (21) can be derived as:

(23) $$\begin{array}{cc} \; & {\dot{V}}_{o}=\frac{1}{2}\left[{\tilde{p}}_{3},{\tilde{p}}_{4}\right]\left(\left[\begin{array}{cc} -l(t)\zeta & \omega \\ -\omega & -l(t)\zeta \end{array}\right]+{\left[\begin{array}{cc} -l(t)\zeta & \omega \\ -\omega & -l(t)\zeta \end{array}\right]}^{T}\right)\left[\begin{array}{c} {\tilde{p}}_{3}\\ {\tilde{p}}_{4} \end{array}\right]\\ \; & \Rightarrow {\dot{V}}_{o}\leq \left[{\tilde{p}}_{3},{\tilde{p}}_{4}\right]\left[\begin{array}{cc} -l(t)\zeta & 0\\ 0 & -l(t)\zeta \end{array}\right]\left[\begin{array}{c} {\tilde{p}}_{3}\\ {\tilde{p}}_{4} \end{array}\right]\\ \; & \Rightarrow {\dot{V}}_{o}\leq -l\left(t\right)\zeta V_{o}. \end{array}$$

Therefore, the estimation error exponentially converges to zero in presence of slowly varying measurement uncertainties.

Case 2: For measurement disturbances which are not slowly varying, the rate of variation is bounded by the constant $c_{2}$ defined in Assumption I. Taking the same Lyapunov function for case 1, and deriving the rate of change of this function along the error dynamics (19), one can obtain:

(24) $${\dot{V}}_{o}\leq \left[{\tilde{p}}_{3},{\tilde{p}}_{4}\right]\left[\begin{array}{cc} -l(t)\zeta & 0\\ 0 & -l(t)\zeta \end{array}\right]\left[\begin{array}{c} {\tilde{p}}_{3}\\ {\tilde{p}}_{4} \end{array}\right]+l\left(t\right)\left[{\tilde{p}}_{3},{\tilde{p}}_{4}\right]\left[\begin{array}{c} {\dot{n}}_{x}\\ {\dot{n}}_{y} \end{array}\right]$$

(25) $$\Rightarrow {\dot{V}}_{o}\leq -l\left(t\right)\zeta V_{o}+l\left(t\right)||\left[\begin{array}{c} {\tilde{p}}_{3}\\ {\tilde{p}}_{4} \end{array}\right]\left|||\right|\left[\begin{array}{c} {\dot{n}}_{x}\\ {\dot{n}}_{y} \end{array}\right]\left|\right|$$

(26) $$\Rightarrow {\dot{\sqrt{V}}}_{o}\leq -l\left(t\right)\frac{\zeta }{2}{\sqrt{V}}_{o}+\frac{l(t)}{{\sqrt{V}}_{o}}\left|\right|\left[\begin{array}{c} {\tilde{p}}_{3}\\ {\tilde{p}}_{4} \end{array}\right]\left|\right|c_{2}$$

(27) $$\Rightarrow {\dot{\sqrt{V}}}_{o}\leq -l\left(t\right)\frac{\zeta }{2}{\sqrt{V}}_{o}+c_{2}\left||l\left(t\right)|\right|$$

Hence, there exists a positive constant $\gamma_{1}$ such that,

(28) $$\Rightarrow \left|\right|\left[\begin{array}{c} {\tilde{p}}_{3}\left(t\right)\\ {\tilde{p}}_{4}\left(t\right) \end{array}\right]\left|\right|\leq exp\left(-tl\left(t\right)\frac{\zeta }{2}\right)\left|\right|\left[\begin{array}{c} {\tilde{p}}_{3}\left(0\right)\\ {\tilde{p}}_{4}\left(0\right) \end{array}\right]\left|\right|+\frac{2\gamma_{1}c_{2}}{\zeta }$$

Therefore, the estimation error is always bounded, and the steady state value is given by $\frac{2\gamma_{1}c_{2}}{\zeta }$. The constant $\gamma_{1}$ depends on the norm of the observer gain l(t).

Selection of $l_{o},l_{a},l_{n}$: Generally in the neighborhood of t≈0 (transient behavior) the variation in measurement uncertainties are smaller compared to the norm of estimation errors $\left|\right|\left[\begin{array}{cc} {\tilde{p}}_{3} & {\tilde{p}}_{4} \end{array}\right]\left|\right|$. By choosing a moderate gain $l_{o}>l_{a}>0$, the estimation error quickly converges to a small value. However, after the initial transients, the estimation error can be smaller than the constant $c_{2}$. So a larger l(t) may amplify the measurement uncertainty. Therefore, the observer gain should be very small for the steady state uncertainty attenuation. We know,

(29) $$\lim_{t\to \infty }l\left(t\right)=l_{a}.$$

So $l_{a}$ should be chosen sufficiently small. The constant $l_{n}$ determines the time evolution of the observer gain l(t). It should not be tuned according to the need. The above analysis can be summarised as the following theorem.

**Theorem** **1.** *Let the assumptions I and II hold true. If an observer is constructed as given in (14), and the observer gain is chosen as (15), then the estimation errors exponentially converge to zero for slow varying measurement uncertainties. Moreover, the observer guarantees estimation error convergence to a small neighborhood of origin for measurement uncertainties satisfying assumption I.*

## 4. Robust Control Law

From the previous section, it can be concluded that the estimation error converges to zero exponentially, or converges to a very small neighborhood of zero. So, it is safe to assume that the estimate $\hat{\Theta }$ also converges to its true value Θ. However, there may be a small deviation for case 2 described in the previous section. Let’s choose a control law

(30) $$U={\hat{\Theta }}^{-1}\left(\xi +\xi_{r}\right)$$

for the mathematical model (7). The variable $\xi ={\left[\xi_{1},\xi_{2}\right]}^{T}$ represents a nominal PD control law, and $\xi_{r}={\left[\xi_{r1},\xi_{r2}\right]}^{T}$ represents the robust term to deal with inaccuracies in $\hat{\Theta },x,y$. As the estimation error is very close to zero,

(31) $$\Theta {\hat{\Theta }}^{-1}=I\left(1\pm \epsilon \right)$$

where *I* is an identity matrix of second-order, and ϵ is a small positive number. Here, the deviation is assumed to be the same in both the diagonal elements. Even though they are different, the controller design remains the same. Rewrite the closed-loop system as:

(32) $$\left[\begin{array}{c} \ddot{x}\\ \ddot{y} \end{array}\right]=\left[\begin{array}{c} \xi_{1}+d_{x}+\xi_{r1}\\ \xi_{2}+d_{y}+\xi_{r2} \end{array}\right]$$

where $d_{x}$ and $d_{y}$ represent the lumped disturbances arising due to the uncertainties present in x,y, and the additional terms $\pm \epsilon \xi_{1},\pm \epsilon \xi_{2}$. The following assumption is made to design the robust part $\xi_{r}$ of the control law.

**Assumption** **3.** *There exist two positive sclars $d_{1}$ and $d_{2}$ such that*

(33) $$\left|\right|d_{x}\left|\right|\leq d_{1},\;\;||d_{y}\left|\right|\leq d_{2}.$$

The assumption III is not conservative since the estimation error is very small, and the measurement errors are bounded from assumption I. From (32), it is clear that the closed-loop dynamics are decoupled. So the control input pairs $\xi_{1},\xi_{r1}$ and $\xi_{2},\xi_{r2}$ can be designed independently.

For this purpose, choose two sliding surfaces:

(34) $$s_{x}={\dot{e}}_{x}+k_{1}e_{x},\;s_{y}={\dot{e}}_{y}+k_{2}e_{y}$$

where $e_{x}=x-x_{d},\;e_{y}=y-y_{d}$, and $k_{1},k_{2}$ are positive scalars. Let’s define a candidate Lyapunov function

(35) $$V_{x}=\frac{1}{2}s_{x}^{2}.$$

The time derivative of $V_{x}$ along (32) is given by

(36) $$\begin{array}{cc} \; & {\dot{V}}_{x}=s_{x}{\dot{s}}_{x}=s_{x}\left(\ddot{x}-{\ddot{x}}_{d}+k_{1}{\dot{e}}_{x}\right)\\ \; & =s_{x}\left(\xi_{1}+\xi_{r1}+d_{x}-{\ddot{x}}_{d}+k_{1}{\dot{e}}_{x}\right) \end{array}$$

Choose $\xi_{1}={\ddot{x}}_{d}-k_{1}{\dot{e}}_{x}-k_{3}s_{x}$ ($k_{3}$ is a positive scalar), hence

(37) $${\dot{V}}_{x}=s_{x}\left(-k_{2}s_{x}+\xi_{r1}+d_{x}\right)$$

choose $\xi_{r1}=-\left(1+d_{1}\right)tanh\left(s_{x}\right)$, then

(38) $$\begin{array}{cc} \; & {\dot{V}}_{x}\leq -k_{3}s_{x}^{2}-\left(1+d_{1}\right)s_{x}tanh\left(s_{x}\right)+d_{1}\left|\right|s_{x}\left|\right|\\ \; & \Rightarrow {\dot{V}}_{x}\leq -k_{3}s_{x}^{2}-\left(1+d_{1}\right)\left|\right|s_{x}\left|\right|+d_{1}\left|\right|s_{x}\left|\right|. \end{array}$$

So the rate of change of $V_{x}$ is negative definite, and $s_{x}\to 0$ asymptotically.

(39) $$s_{x}\to 0\Rightarrow {\dot{e}}_{x}=-k_{1}e_{x}\Rightarrow x\to x_{d}.$$

A similar analysis can be done for the sliding function $s_{y}$. The controller design procedure can be summarized in the following theorem.

**Theorem** **2.** *Let all the assumptions be satisfied. If the observer design is done according to Theorem 1, and the control law is chosen as*

(40) $$\begin{array}{cc} \; & \xi_{1}={\ddot{x}}_{d}-k_{1}{\dot{e}}_{x}-k_{3}s_{x},\;\xi_{r1}=-\left(1+d_{1}\right)tanh\left(s_{x}\right)\\ \; & \xi_{2}={\ddot{y}}_{d}-k_{2}{\dot{e}}_{x}-k_{4}s_{x},\;\xi_{r2}=-\left(1+d_{2}\right)tanh\left(s_{y}\right)\\ \; & \left[\begin{array}{c} f\\ \omega \end{array}\right]={\hat{\Theta }}^{-1}\left[\begin{array}{c} \xi_{1}+\xi_{r1}\\ \xi_{2}+\xi_{r2} \end{array}\right]\\ \; & v=\zeta \end{array}$$

*then the trajectory tracking errors $e_{x},e_{y}$ asymptotically converge to zero.*

Selection of $\xi_{1}\left(0\right)$: To assure perfect trajectory tracking, one should choose $\xi_{1}\left(o\right)=\sqrt{x{\left(0\right)}^{2}+y{\left(0\right)}^{2}}$, which simplifies the control law into a feed-forward action. This also avoid zero crossing of *v*, which may create problem in observer convergence and stability in a closed-loop. Another effective way to avoid this problem is to reset $\xi_{1}\left(t\right)$, whenever it is very near to zero.

## 5. Simulation Results and Discussion

To verify the efficacy of the proposed approach, the closed-loop simulations were carried out for different cases. The path tracking task was common in all the cases, where the desired path was to track a circle of 2 m radius. For this purpose, the desired commands for X and Y positions are given as $x_{d}=2sin\left(t\right),y_{d}=2cos\left(t\right)$.

The distance between wheels ($L_{2}$) was assumed to be 0.4 units, the length of the robot ($L_{1}$) was considered as 0.7 units, and the wheels were assumed to be placed at an angle (α) 30 degrees from the COG. The observer gains could be chosen to be any positive number as discussed in Section 3. However, care should be taken such that the observer could assure robust performance in the presence of both slow and fast varying uncertainties. After some trial and error, the design parameters of time-varying observer gain were chosen as $l_{o}=1.1,l_{a}=0.2,l_{n}=0.7$, i.e.,

(41) l(t)=0.9exp(−0.7t)+0.2.

The controller parameters should be chosen such that the transient and steady state performance are satisfactory. This could be ensured by analyzing the poles in closed loop, or tuning the gains till a satisfactory response was obtained. The design parameters for the sliding mode controller were chosen as $k_{1}=k_{2}=3,\;k_{3}=k_{4}=2.5$.

The initial condition of the robot COG was chosen as (3,1). To ensure perfect trajectory tracking the initial condition for the dynamic integrator was chosen as $\xi_{1}\left(0\right)=\sqrt{10}$.

**Figure 2:**
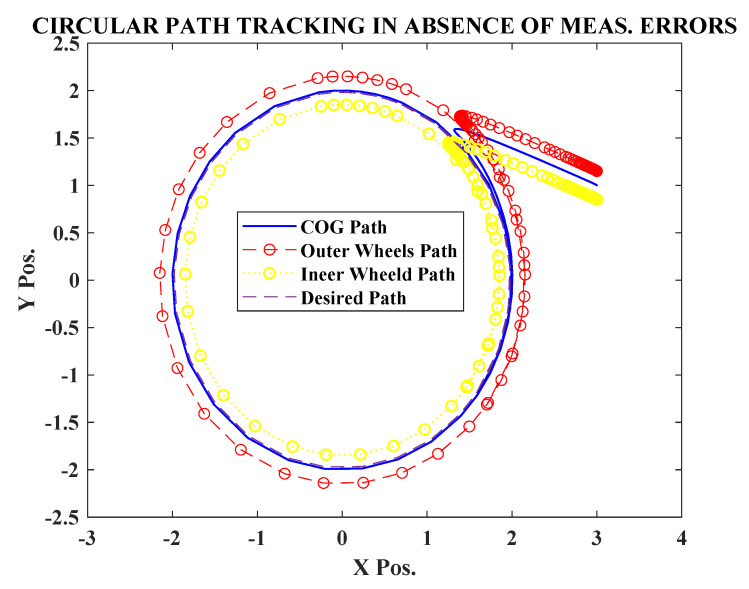
Circular path tracking without sensor errors.

**Figure 3:**
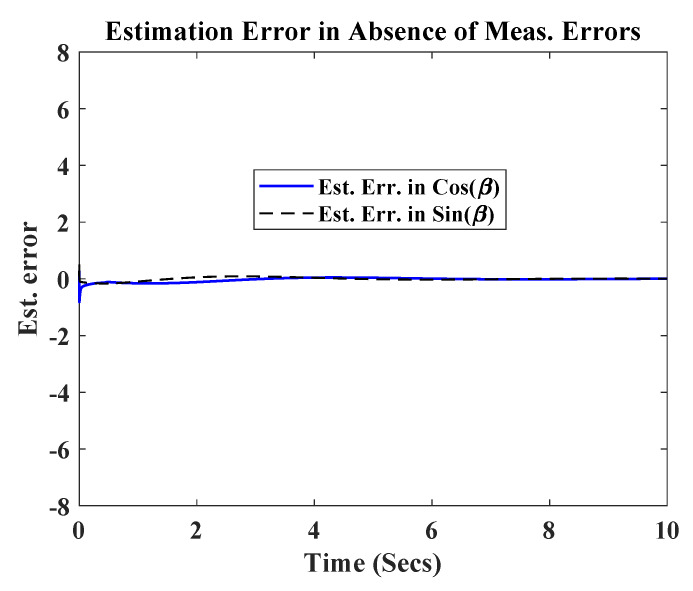
Estimation error in absence of measurement errorsors.

Initially the simulation was carried out in the absence of any measurement errors $n_{x}=n_{y}=0$. It can be observed from Figure 2 that the robot was able to successfully track the circular path, starting from an initial position (x(0),y(0))=(3,1). The observer also efficiently estimated the signals cos(β) and sin(β), after an initial transient. Figure 3 shows, the transients were well within maximum bounds for cos(β) and sin(β).

**Figure 4:**
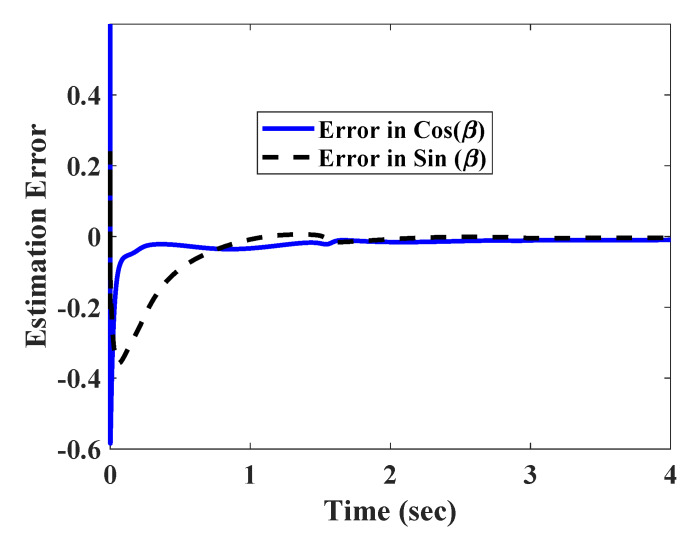
Estimation error in presence of slow varying of measurement errors.

**Figure 5:**
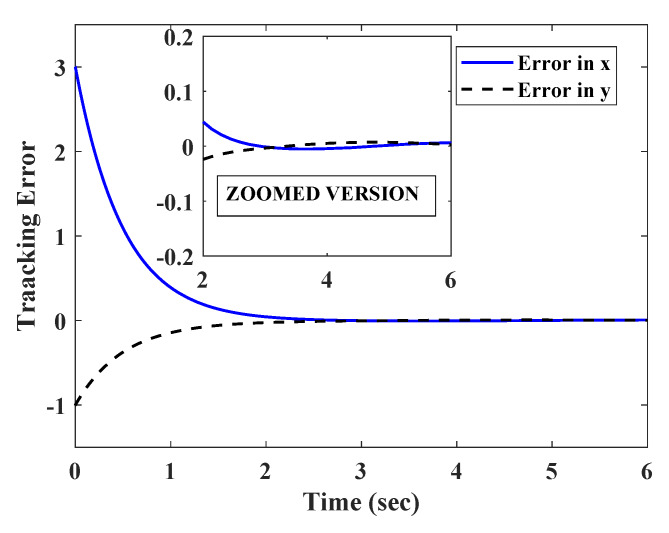
Convergence of X and Y position of robot in presence of slow varying measurement errors.

**Figure 6:**
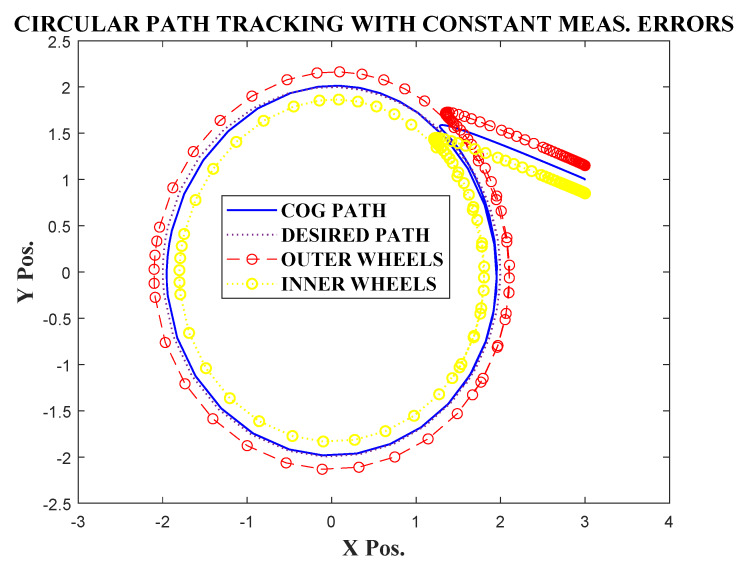
Circular path tracking with slow varying measurement. errors.

The same tracking task was given again with corrupting the x and y measurements by a set of slow varying signals $n_{x}=0.3tanh\left(x\right),n_{y}=0.2*tanh\left(y\right)$. It should be noted that, the measurement disturbances $n_{x},n_{y}$ satisfied assumption 1. The estimation error, trajectory convergence, and path tracking are given in Figure 4, Figure 5 and Figure 6.

It can be observed from Figure 6 that the COG of the robot had a small discrepancy with the desired path. This can be explained by the small errors in the convergence of x and y state trajectories (Figure 5). It should also be noted that the estimation errors (Figure 4) converged to a very small bound after initial transients.

To test the robustness of the technique against fast varying measurement errors, the position in x and y coordinates were corrupted with a set of fast varying signals $n_{x}=0.3sin\left(10t\right),n_{y}=0.25cos\left(10t\right)$, which satisfied assumption 1.

**Figure 7:**
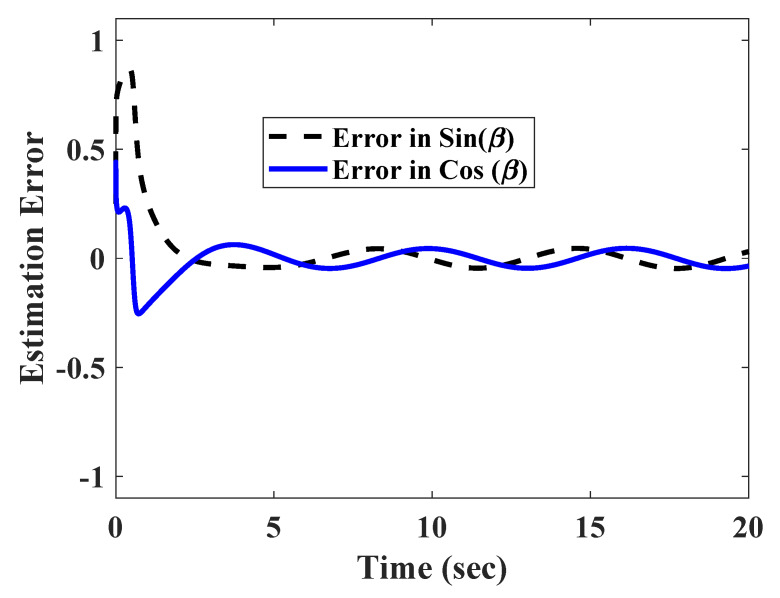
Estimation error in presence of fast varying of measurement errors.

**Figure 8:**
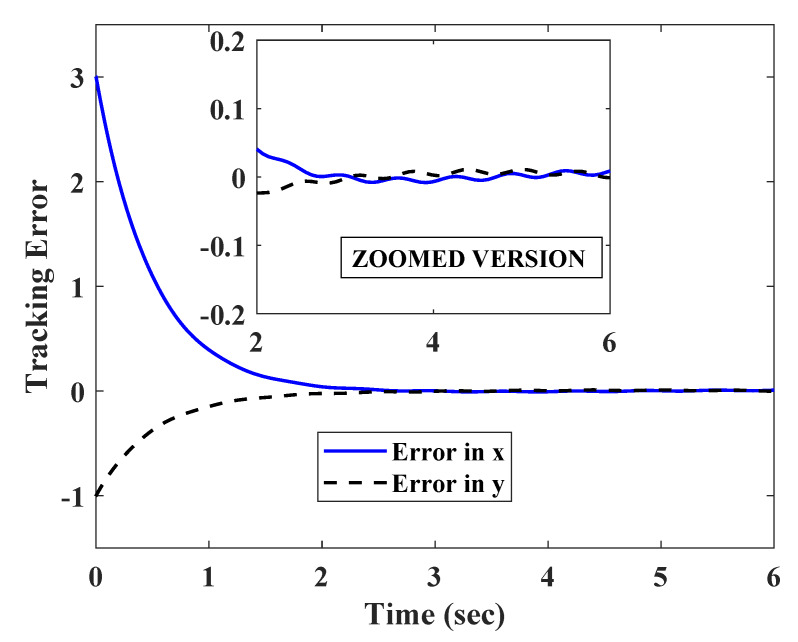
Convergence of X and Y position of robot in presence of fast varying measurement errors.

**Figure 9:**
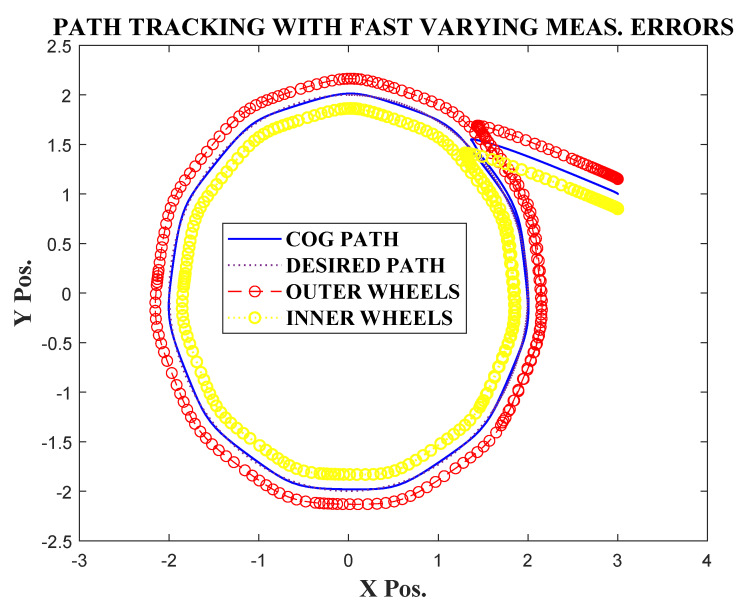
Circular path tracking with fast varying measurement errors.

The estimation errors shown in Figure 7 point to the fact that the observer was able to attenuate the effect of the uncertainties to a large extent. However, a small oscillating error still remained in the steady state. For this reason, the trajectories converged to a small steady state error, as shown in Figure 8. As the trajectories oscillate with a very small magnitude in the steady state, the path tracking also result in a very small error (as shown in Figure 9).

**Figure 10:**
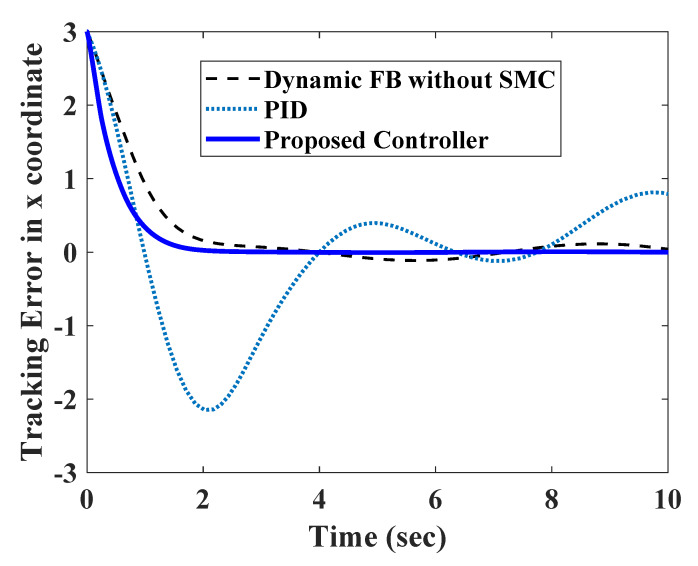
Comparison of position error in x direction.

**Figure 11:**
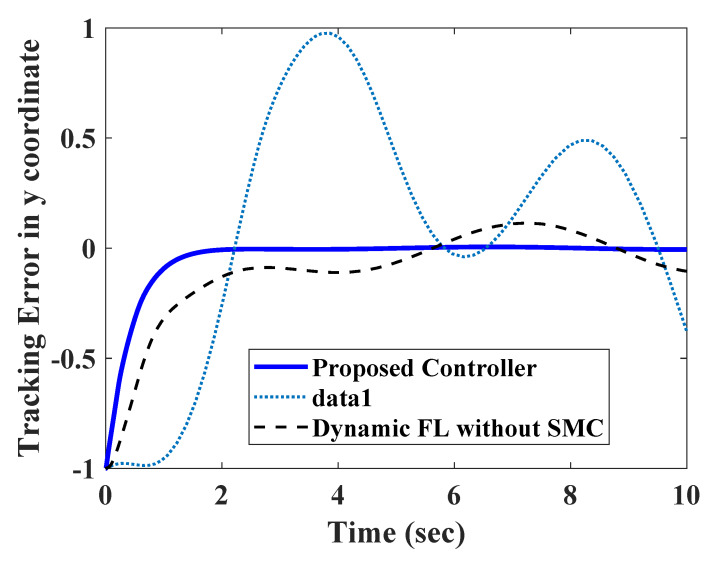
Comparison of Position Error in y direction.

To test the efficacy of the proposed controller, it was compared with a conventional PID and a dynamic feedback linearization strategy without the sliding mode controller (SMC). The tracking errors for all three controllers are shown in Figure 10 and Figure 11. The PID controller took a longer time to settle down, and had comparatively larger transients. As the measurements were corrupted with disturbances, they were amplified by the derivative part of the control law. A better performance could be expected from PI control law, but it could not assure transient performance in nonlinear systems. The dynamic feedback linearization without SMC was able to assure a bounded tracking performance. However, the steady state errors were comparatively larger due to the absence of any robust part in the controller.

## 6. Conclusions

A new robust observer-based control strategy is developed for car-like robots in endogenous environments, where accurate measurements for robot pose is difficult to obtain. The proposed approach can simultaneously deal with uncertainties in position measurement and absence of heading angle information. A robust observer with time varying gain was used to directly estimate the trigonometric functions involving heading angles. The estimates were exploited to formulate a dynamic feedback linearization based sliding mode design. The Lyapunov stability analysis assures closed-loop stability, and simulation results show the efficacy of the proposed methodology. The controller does not account for any modeling uncertainties and friction effects, which are the subjects of the future works.

## Abbreviations

α: Orientation angle of wheel with COG, β: Heading angle, *v*: Linear velocity of COG, ω: Angular velocity of COG, ζ: State of the dynamic compensator, Θ: Head angle matrix, ξ: Control input for linearized system, $\hat{p}$: Estimate of p, $\tilde{p}$: Estimation error in p, ϵ: Small constant for tolerance in controller.

## References

[1] Markets and Markets (2015). Cleaning Robot Market by Product. http://www.marketsandmarkets.com/Market-Reports/cleaning-robot-market-22726569.html.

[2] Liu K., Wang C. (2013). A technical analysis of autonomous floor cleaning robots based on us granted patents. Eur. Int. J. Sci. Technol..

[3] Hayat A.A., Parween R., Elara M.R., Parsuraman K., Kandasamy P.S. (2019). Panthera: Design of a reconfigurable pavement sweeping robot. Proceedings of the 2019 International Conference on Robotics and Automation (ICRA).

[4] Le A.V., Nhan N.H.K., Mohan R.E. (2020). Evolutionary Algorithm-Based Complete Coverage Path Planning for Tetriamond Tiling Robots. Sensors.

[5] Hayat A.A., Elangovan K., Rajesh Elara M., Teja M.S. (2018). Tarantula: Design, Modeling, and Kinematic Identification of a Quadruped Wheeled Robot. Appl. Sci..

[6] Tun T.T., Huang L., Mohan R.E., Matthew S.G.H. (2019). Four-wheel steering and driving mechanism for a reconfigurable floor cleaning robot. Autom. Constr..

[7] Manimuthu A., Vu L.A., Mohan R.E., Veerajagadeshwar P., Khanh Nhan N.H., Ku P.-C. (2019). Energy Consumption Estimation Model for Complete Coverage of a Tetromino Inspired Reconfigurable Surface Tiling Robot. Energies.

[8] Rayguru M.M., Roy S., Kar I.N. (2019). Time-Scale Redesign-Based Saturated Controller Synthesis for a Class of MIMO Nonlinear Systems. IEEE Transactions on Systems, Man, and Cybernetics: Systems.

[9] Yin J., Apuroop K.G.S., Tamilselvam Y.K., Mohan R.E., Ramalingam B., Le A.V. (2020). Table Cleaning Task by Human Support Robot Using Deep Learning Technique. Sensors.

[10] Veerajagadheswar P., Sivanantham V., Devarassu M., Elara M.R. (2019). Htetran—A Polyabolo Inspired Self Reconfigurable Tiling Robot. Proceedings of the 2019 IEEE/RSJ International Conference on Intelligent Robots and Systems (IROS).

[11] Parween R., Shi Y., Parasuraman K., Vengadesh A., Sivanantham V., Ghanta S., Mohan R.E. (2019). Modeling and Analysis of hHoneycomb—A Polyhex Inspired Reconfigurable Tiling Robot. Energies.

[12] Yanagida T., Elara Mohan R., Pathmakumar T., Elangovan K., Iwase M. (2017). Design and Implementation of a Shape Shifting Rolling–Crawling–Wall-Climbing Robot. Appl. Sci..

[13] Le A.V., Arunmozhi M., Veerajagadheswar P., Ku P.C., Minh T.H.Q., Sivanantham V., Mohan R.E. (2018). Complete path planning for a tetris-inspired self-reconfigurable robot by the genetic algorithm of the traveling salesman problem. Electronics.

[14] De Oliveira H.F.P., de Sousa A.J.M., Moreira A.P.G.M., da Costa P.J.C.G. (2008). Dynamical models for omni-directional robots with 3 and 4 wheels. Proceedings of the 5th International Conference on Informatics in Control, Automation and Robotics.

[15] De Luca A., Oriolo G., Vendittelli M. (2001). Control of wheeled mobile robots: An experimental overview. Ramsete.

[16] Lee S.O., Cho Y.J., Hwang-Bo M., You B.J., Oh S.R. A stable target-tracking control for unicycle mobile robots. Proceedings of the 2000 IEEE/RSJ International Conference on Intelligent Robots and Systems (IROS 2000) (Cat. No. 00CH37113).

[17] Rayguru M., Kar I. (2019). Contraction theory approach to disturbance observer based filtered backstepping design. J. Dyn. Syst. Meas. Control.

[18] Zhou H., Chen R., Zhou S., Liu Z. (2019). Design and Analysis of a Drive System for a Series Manipulator Based on Orthogonal-Fuzzy PID Control. Electronics.

[19] Mu J., Yan X., Jiang B., Spurgeon S.K., Mao Z. Sliding mode control for a class of nonlinear systems with application to a wheeled mobile robot. Proceedings of the 54th IEEE Conference on Decision and Control (CDC).

[20] Cheng X., Tu X., Zhou Y., Zhou R. (2019). Active Disturbance Rejection Control of Multi-Joint Industrial Robots Based on Dynamic Feedforward. Electronics.

[21] Acho L. (2017). Event-Driven Observer-Based Smart-Sensors for Output Feedback Control of Linear Systems. Sensors.

[22] Cui M. (2019). Observer-Based Adaptive Tracking Control of Wheeled Mobile Robots With Unknown Slipping Parameters. IEEE Access.

[23] Boukhari M., Chaibet A., Boukhnifer M., Glaser S. (2018). Proprioceptive Sensors’ Fault Tolerant Control Strategy for an Autonomous Vehicle. Sensors.

[24] Hao Y., Wang J., Chepinskiy S.A., Krasnov A.J., Liu S. Backstepping based trajectory tracking control for a four-wheel mobile robot with differential-drive steering. Proceedings of the 36th Chinese Control Conference (CCC).

[25] De Wit C.C., Bastin G., Siciliano B. (1996). Theory of Robot Control.

